# Seronegative limbic encephalitis manifesting as subacute amnestic syndrome: a case report and review of the literature 

**DOI:** 10.1186/s13256-021-02742-4

**Published:** 2021-03-24

**Authors:** Ismail Ibrahim Ismail, Fahad Alnaser, Jasem Y. Al-Hashel

**Affiliations:** 1 Department of Neurology, Ibn Sina Hospital, Sabah Medical Region, Kuwait; 2 Department of Radiology, Ibn Sina Hospital, Sabah Medical Region, Kuwait; 3 Health Sciences Centre, Department of Medicine, Kuwait University, Jabriya, Kuwait

**Keywords:** Limbic encephalitis, Seronegative, Autoimmune, Amnesia, Memory

## Abstract

**Background:**

Limbic encephalitis (LE), a variant of autoimmune encephalitis, is inflammation of the limbic system of the brain. The disorder presents with subacute impairment of short-term memory, psychiatric manifestations, confusion and seizures. “Seronegative LE” is a challenging diagnosis in the absence of well-characterized autoantibodies.

**Case presentation:**

A 33-year-old Kuwaiti woman with no relevant past history presented with subacute progressive amnesia of 6-month duration. Magnetic resonance imaging (MRI) showed bilateral hyperintensity of the limbic structures. An extensive workup of the blood and cerebrospinal fluid (CSF) failed to identify paraneoplastic or autoimmune antibodies. The diagnosis of seronegative LE was made, and immunotherapy was initiated, with improvement in cognitive function.

**Conclusion:**

Seronegative LE is a challenging diagnosis. Inability to detect autoantibodies, especially early in the disease course, should not rule out the diagnosis of autoimmune encephalitis. Early diagnosis and treatment with immunotherapy may prevent irreversible brain damage.

## Background

Limbic encephalitis (LE) is an acute noninfectious inflammation of the brain affecting the limbic system. The classical clinical presentation can include amnesia, behavioral changes, psychiatric symptoms, seizures and disturbed level of consciousness [1].

Brierley and colleagues first described the clinical and pathological findings of LE in three patients in 1960 [2], with evidence of inflammation of the limbic structures, particularly the hippocampus and amygdala. This disease generally has two underlying etiologies, paraneoplastic and autoimmune. LE diagnosis requires radiological or pathological evidence of inflammation of the medial temporal lobes and the presence of well-characterized autoantibodies. Seronegative LE is a subgroup of encephalitis with suspected immunological pathology in the absence of any identifiable pathogenic autoantibodies [3].

Despite being previously reported in literature, it is still underdiagnosed, and management is usually delayed for months. Herein, we report a case of a 33-year-old woman who presented with subacute amnesia secondary to seronegative LE. We augmented our case with an updated review of the literature.

## Case presentation

A 33-year-old Kuwaiti woman, with no relevant past medical or psychiatric history, presented to our clinic with a 6-month history of progressive memory loss. Initially, the patient developed subacute onset of short-term memory loss that was mainly noticed by her family and coworkers. The condition was associated with depressed mood, insomnia, crying attacks and back pain, with no other associated neurological complaints. She was evaluated by a local psychiatrist who diagnosed her with major depressive disorder (MDD), and started her on antidepressant medication (vortioxetine 10 mg/day). Within the next month, the dose was increased to 20 mg per day and a benzodiazepine (alprazolam 0.5 mg/day) was added. The depressed mood and insomnia showed improvement, but her memory loss progressed.

The patient was referred to a neurologist 3 months after onset. Her neurological examination was reported as normal apart from short-term memory loss. Her Mini-Mental State Examination (MMSE) score was 23/30. Magnetic resonance imaging (MRI) of the brain was requested and showed a bilateral asymmetric hyperintense signal of both hippocampi on T2-weighted and fluid-attenuated inversion recovery (FLAIR) images, without contrast enhancement. Cerebrospinal fluid (CSF) analysis revealed lymphocytic pleocytosis with glucose and protein within the normal range. Virology screening for herpes simplex virus (HSV) was negative. However, the patient received acyclovir 10 mg/kg intravenously every 8 hours for 14 days with no improvement.

We evaluated the patient 6 months after symptoms onset. Her memory loss had progressed since her last evaluation. However, she had not developed any seizures or other neurological or psychological manifestations. Her vital signs and general examination were normal. Her neurological examination showed a conscious and oriented patient with normal speech. Her memory assessment showed severe anterograde, and to a lesser extent retrograde, short-term memory loss, with MMSE of 20/30 and a Montreal Cognitive Assessment (MoCA) score of 21/30. Otherwise, her motor, sensory and cerebellar examination were normal.

**Fig. 1:**
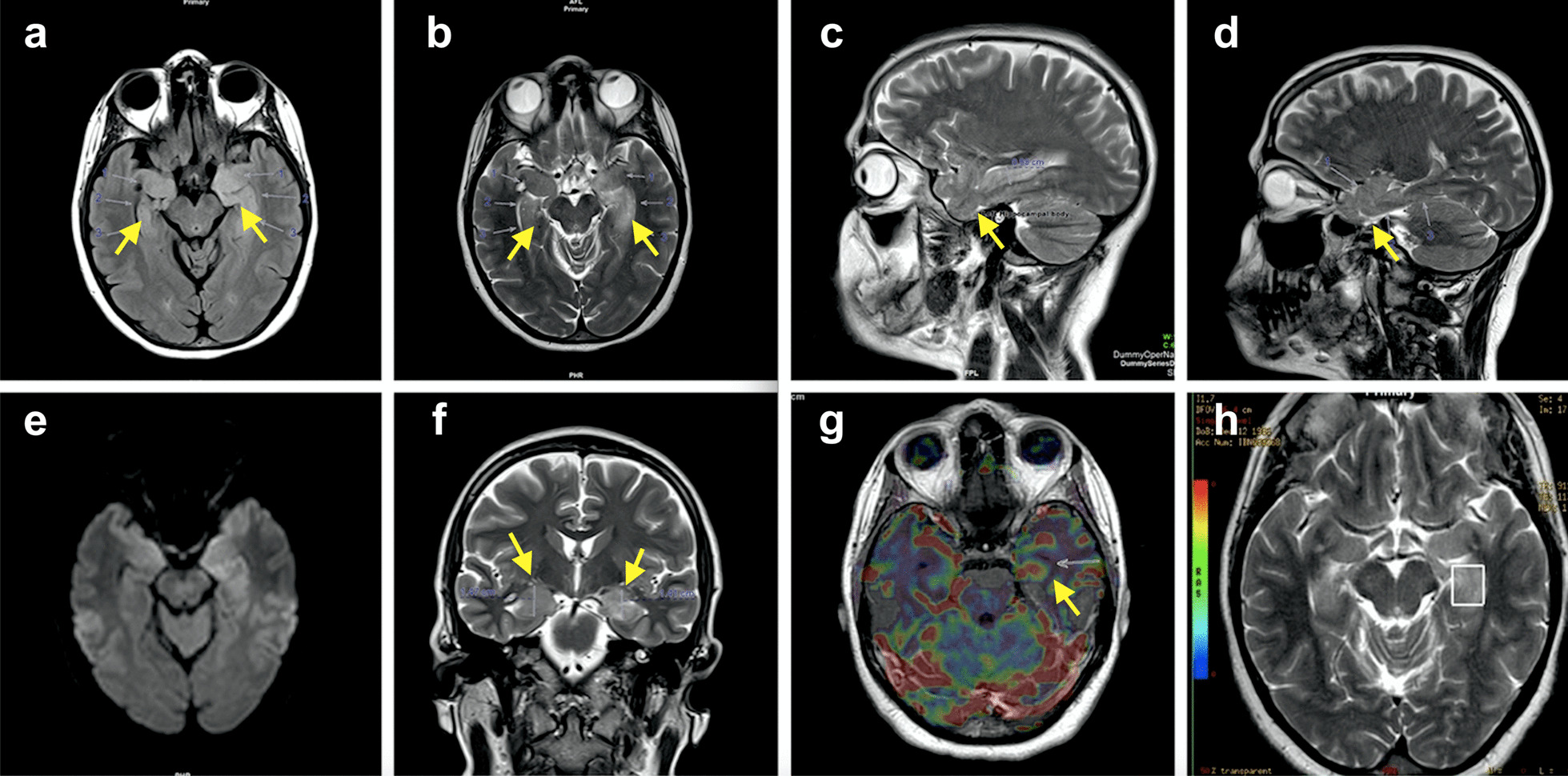
Magnetic resonance imaging findings. **a** Axial fluid-attenuated inversion recovery (FLAIR), **b** axial T2-weighted images, showing bilateral hyperintense signals and hypertrophy of medial temporal lobes and hippocampi, **c**, **d** sagittal T2-weighted images showing hypertrophy of hippocampi (left: head thickness 11 mm, body thickness 9.8 mm, right: head thickness 15 mm, body thickness 10 mm) and amygdala bilaterally (arrows), **e** diffusion-weighted imaging showing no restriction, **f** coronal T2-weighted images showing bilateral hippocampal hypertrophy (arrows), **g** magnetic resonance perfusion images showing a focal increase in regional cerebral blood flow on the left side (arrow), and **h** magnetic resonance spectroscopy showing increased Cho/NAA ratio on the left side

A follow-up brain MRI (Fig. 1) showed bilateral hyperintensity and hypertrophy of the head, body and tail of the bilateral hippocampi (more on the left side), and amygdala bilaterally. There was no associated contrast enhancement or restriction in diffusion-weighted image (DWI) sequences. However, there was a focal increase in the regional cerebral blood flow (rCBF) on the left side in perfusion imaging, and an increase in the choline peak (increased choline/*N*-acetylaspartate [Cho/NAA] ratio) on magnetic resonance spectroscopy (MRS).

Electroencephalography (EEG) showed electrographic seizure activity manifested as temporal intermittent rhythmic delta activity (TIRDA) appearing from the right inferior, frontal and opercular regions, with frequent right anterior–mid-temporal spikes and spike-wave discharges.

A comprehensive blood workup showed normal complete blood count (CBC), renal and liver function, serum electrolytes, inflammatory markers (erythrocyte sedimentation rate [ESR], C-reactive protein), serum vitamins B1, B6, B12 and folate, protein electrophoresis, immunoglobulin essay, thyroid function and antithyroid autoantibodies. Serology for HSV, hepatitis B and C, human immunodeficiency virus (HIV), Lyme disease, syphilis and *Toxoplasma* were negative. A panel for vasculitis including rheumatoid factor (RF), antinuclear antibody (ANA), anti-double-stranded DNA antibody (anti-dsDNA), extractable nuclear antigen (ENA) and antineutrophil cytoplasmic antibodies (ANCA) was also negative.

Extensive workup for autoimmune antibodies associated with LE was performed including anti-*N*-methyl-d-aspartate (NMDA) receptor, α-amino-3-hydroxy-5-methyl-4-isoxazolepropionic acid (AMPA) receptor, voltage-gated potassium channels (VGKC), leucine-rich glioma-inactivated protein 1 (LGI-1), contactin-associated protein-like 2 (CASPR2), gamma-aminobutyric acid-B (GABA-B), glutamic acid decarboxylase (GAD) and dipeptidyl peptidase-like protein-6 (DPPX) antibodies, and it yielded negative results.

A paraneoplastic workup including anti-Yo, anti-Hu, anti-Ri, anti Ma1/2, CEA19.9, CA125, CA15.3 and anti-amphiphysin, collapsin response-mediator protein-5 (CRMP5) was also negative.

Lumbar puncture was repeated, and CSF analysis showed lymphocytic pleocytosis (17 cells/mm^3^) with normal glucose (4.25 mol/L) and protein (362 mg/L). Polymerase chain reaction virology screening for neurotropic viruses (HSV, varicella-zoster virus, Epstein–Barr virus, cytomegalovirus) was negative. It also tested negative for bacterial, fungal or mycobacterial infection. Anti-VGKC, NMDA and AMPA receptor antibodies were negative, but oligoclonal bands (OCB) were positive.

Computed tomography of the chest, abdomen and pelvis, and whole-body positron emission tomography (PET) were performed and showed no evidence of malignancy.

The patient received a diagnosis of “seronegative LE” and was treated with a course of intravenous methylprednisolone 1000 mg per day for 5 days, followed by 1000 mg once weekly for 8 weeks. Lacosamide 50 mg twice daily was added after the abnormal EEG findings.

**Fig. 2:**
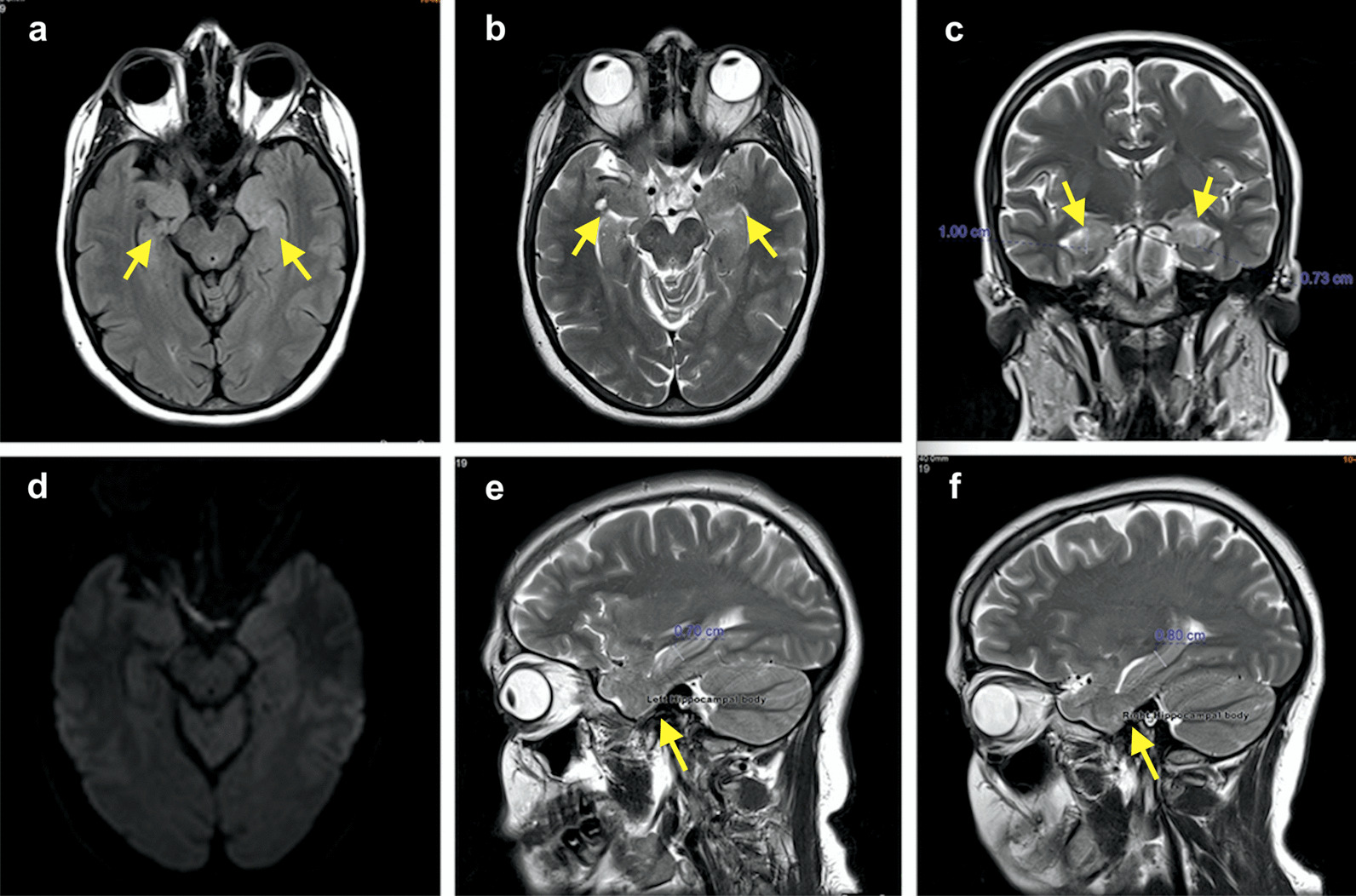
Follow-up magnetic resonance imaging after 2 months of immunotherapy. **a** Axial fluid-attenuated inversion recovery (FLAIR), **b** axial T2-weighted images, **c** coronal T2-weighted images, **e**, **f** sagittal T2-weighted images, showing decrease in the T2/FLAIR high signal intensity of the hippocampi bilaterally, and regression of the hypertrophy of hippocampi (left: head thickness 7 mm, body thickness 8.5 mm, right: head thickness 10 mm, body thickness 8 mm) and amygdala bilaterally (arrows), **d** diffusion-weighted imaging showing no diffusion restriction.

A follow-up brain MRI was performed after 2 months of therapy, and showed decreased hyperintensity and hypertrophy of the limbic structures (Fig. 2).

**Fig. 3:**
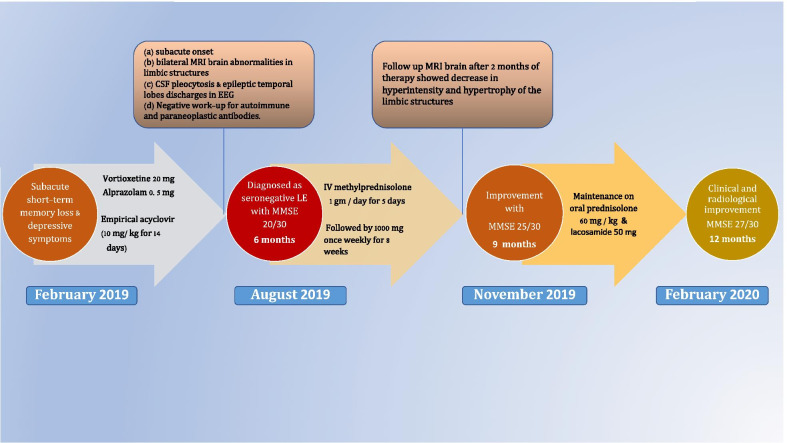
Timeline of the clinical course

She was maintained on oral prednisolone (60 mg/day) that was tapered gradually over 6 months. The patient showed subjective and objective improvement in her memory. MMSE score was 23/30 after 1 month, 25/30 after 3 months and 27/30 after 6 months. A timeline of the clinical course is highlighted in Fig. 3.

## Discussion

Seronegative LE is a challenging diagnosis. It requires a classical clinical presentation, radiological evidence of limbic system and medial temporal lobe affection, and the absence of pathogenic autoantibodies or evidence of malignancy [4].

In our report, the patient presented with subacute progressive amnesia and depression, bilateral hippocampal hyperintensity and hypertrophy on MRI, abnormal EEG, lymphocytic pleocytosis and positive OCB in CSF, and negative screening for autoantibodies in blood and CSF. Improvement in memory on immunosuppressive therapy further supported the diagnosis.

LE is a variant of encephalitis that affects the medial temporal lobe and hippocampus. It is usually classified as autoimmune or paraneoplastic. Autoantibodies are directed against neuronal cell membrane surfaces, intracellular antigens or synaptic antigens, with overlapping clinical and imaging features [5]. Symptoms of LE include subacute onset, usually in weeks, of short-term memory loss, depression, anxiety, delusions, hallucinations, confusion, behavioral changes and seizures. It affects men more than women (2:1) around the age of 40 years [6]. It is estimated that around 7–26% of all autoimmune LE cases present without detectable antibodies [7, 8].

Despite being previously reported in literature, LE it is still underdiagnosed, and management is usually delayed. This was recently highlighted in the gripping memoir *Brain on Fire: My Month of Madness* by Susannah Cahalan [9], which was adapted into a film of the same name. The female journalist from New York described her experience with anti-NMDA receptor encephalitis that was dominated by presentation of delusions, hallucinations, and later with neurological symptoms. Moreover, the misdiagnosis of those cases as herpetic encephalitis as a result of specific predilection for medial temporal lobes further adds to the morbidity and exposes patients to the dangers of unnecessary medications [10].

Several antibodies have been associated with immune-mediated LE. The clinical and radiological features do not reliably differentiate seropositive from seronegative LE, or paraneoplastic from non-paraneoplastic LE [11]. However, certain features have been found to be more commonly associated with one or the other autoantibody: NMDA receptor-associated LE usually presents with seizures and psychiatric symptoms in association with ovarian teratoma in young women, VGKC antibody-positive LE presents with hyponatremia, faciobrachial dystonic seizures, amnesia and autonomic symptoms in middle-aged men, and GABA-B receptor encephalitis usually presents with psychosis, sleep disturbance and seizures in older men [12, 13].

The diagnosis of seronegative LE is a dilemma to most neurologists, with a wide spectrum of nonspecific clinical manifestations, in the absence of autoantibodies. The necessity of autoantibody detection was found to be impractical, as tests are not readily available in all centers, and results can take several weeks to obtain, delaying the diagnosis. A panel of experts proposed a practical syndrome-based approach to diagnosing autoimmune LE. The proposed criteria for diagnosis of seronegative LE [6] require all four of the following to have been met: (a) subacute onset (less than 3 months) of cognitive deficits, seizures or psychiatric symptoms, (b) bilateral brain abnormalities in medial temporal lobes in T2-weighted MRI images, (c) CSF pleocytosis (more than 5 cells/mm^3^) or EEG with epileptic discharges or slow-wave activity involving the temporal lobes, and (d) reasonable exclusion of alternative causes. All of these criteria were met in our patient.

Furthermore, it should be emphasized that the diagnosis of seronegative LE cannot be made with absolute certainty. First, a paraneoplastic origin can take up to 4–5 years to manifest clinically. Second, there are probably more autoimmune antibodies yet to be discovered, especially with the emergence of more sensitive techniques to detect antibodies that have not been previously identified [14].

MRI is abnormal in around 75% of LE cases. The most common abnormalities are unilateral or asymmetrical bilateral hyperintense signals in FLAIR and T2-weighted images of the medial temporal lobes [11]. Fluorodeoxyglucose (FDG)PET scan is a more sensitive tool, and was found to yield abnormal results in 85% of patients with autoimmune encephalitis. Brain region hypometabolism is most commonly observed early in the disease course [15]. Perfusion studies can also demonstrate abnormalities even before the lesions are identifiable on conventional MRI sequences. The main findings are increased rCBF and cerebral blood volume (CBV), probably due to loss of cerebral vascular autoregulation [16].

CSF analysis shows mild-to-moderate lymphocytic pleocytosis (usually < 100 WBC/mm^3^) in 60–80% of cases, and OCBs are positive in almost 50% of cases. However, its main importance comes from ruling out other causes of LE, and normal basic CSF findings should not lead to a decision against testing for antibodies [17]. EEG is usually abnormal, yet nonspecific. Generalized slowing, temporal lobe epileptiform activity, and unilateral or bilateral predominantly frontotemporal slow-wave activity support the diagnosis [18]. The frequency of seizures is lower than 8% in seronegative LE, compared to more than 50% in antibody-positive LE [19].

The treatment of LE is based on immunosuppressive therapy, and more than 50% of patients show substantial neurological improvement. There are no randomized controlled trials (RCTs) for treatment of LE, and most evidence is either anecdotal or from case series. Moreover, there is no specific treatment protocol for seronegative LE, and it is usually treated as seropositive [20]. The first-line treatment is intravenous administration of corticosteroids (methylprednisolone 1000 mg/day for 5 days followed by 1000 mg once weekly for 8 weeks). High-dose oral prednisolone in a dose of 1 mg/kg per day can also be given after pulse therapy (level 3 evidence). Intravenous immunoglobulin (0.4 g/kg/day for 5 days) and plasmapheresis can be used as first-line treatment if steroids are contraindicated or no improvement is seen on steroids [21]. Early treatment with corticosteroids within 2 months often shows clinical improvement and reduced seizure frequency. Delayed treatment can show variable response, and probably permanent defects. There are no guidelines regarding treatment duration. Oral prednisolone can be tapered gradually over a 6-month period [3]. However, some authors advise continuing for at least 1 or 2 years [22].

Second-line treatments are steroid-sparing agents: rituximab, cyclophosphamide, azathioprine and mycophenolate mofetil. However, there is little evidence supporting their efficacy [23]. Epilepsy should be aggressively treated with antiepileptic drugs, and antidepressants or antipsychotics might be needed [24].

Patients may relapse and should receive appropriate follow-up care. Serial brain MRI scans, regular cognitive assessment and EEG can be used for follow-up to monitor clinical response [25]. Clinical symptoms of relapses can be milder and can occur after many years, and generally warrant the use of second-line treatments [26].

## Conclusion

Seronegative LE is a challenging diagnosis of possible autoimmune etiology.

Inability to detect autoantibodies should neither delay treatment nor rule out the diagnosis. Early diagnosis relies on a high index of suspicion, in spite of negative autoantibodies, especially in those presenting with primarily psychiatric symptoms. Moreover, early initiation of immunotherapy may stop the immunological process and prevent irreversible brain damage and atrophy of the temporal lobes. RCTs are needed to guide treatment strategies for this potentially treatable disorder.

## Data Availability

All original data are available from the corresponding author.
